# Quercetin and its role in biological functions: an updated review

**DOI:** 10.17179/excli2018-1538

**Published:** 2018-08-27

**Authors:** Jae Kwang Kim, Sang Un Park

**Affiliations:** 1 Division of Life Sciences and Convergence Research Center for Insect Vectors, Incheon National University, Incheon 22012, Korea; 2 Department of Crop Science, Chungnam National University, 99 Daehak-ro, Yuseong-gu, Daejeon, 34134, Korea

## ⁯

Dear Editor,

Quercetin is an important flavonol among the members of six subclasses of flavonoid compounds. The name quercetin was derived from *quercetum* (after *Quercus*, i.e., oak), and has been used since 1857 (Fischer et al., 1997[21]). It has been named as 3,3′,4′,5,7-pentahydroxyflavone by the International Union of Pure and Applied Chemistry (IUPAC). It is also known by its synonym 3,3′,4′,5,7-pentahydroxy-2-phenylchromen-4-one (Li et al., 2016[33]). Quercetin is the most widely distributed and extensively studied flavonoid found in various food sources, including fruits, vegetables, nuts, wine, and seeds (Oboh et al., 2016[44]). Quercetin has various biological properties, including antioxidant, anti-inflammatory, antibacterial, antiviral, radical-scavenging, gastroprotective, and immune-modulatory activities (Anand David et al., 2016[6]; Massi et al., 2017[39]). In addition, in several recently-filed patents the wide therapeutic applications of quercetin and its derivatives have been described in detail (Chen et al., 2016[15]; Eid and Haddad, 2017[19]; Sharma et al., 2018[50]). 

Quercetin exhibits a wide range of biological activities and therapeutic applications, which are of interest to the pharmaceutical, cosmetic, and food industries (Biler et al., 2017[10]). Here, we summarize the recent studies that have evaluated the biological and pharmacological activities of quercetin (Table 1(Tab. 1); References in Table 1: Abdelhalim et al., 2018[1]; Afifi et al., 2018[2]; Aghapour et al., 2018[3]; Ahmed et al., 2018[4]; Al-Asmari et al., 2018[5]; Ansar et al., 2016[7]; Atef et al., 2017[8]; Beghoul et al., 2017[9]; Calgarotto et al., 2018[11]; Chan et al., 2018[12]; Chang et al., 2017[13]; Chen et al., 2017[14]; Damiano et al., 2018[16]; Dong et al., 2017[17]; Duranti et al., 2018[18]; Esrefoglu et al., 2017[20]; Funakoshi et al., 2017[22]; Guo et al., 2017[23]; He et al., 2016[24]; Huang et al., 2017[25]; Jeon et al., 2017[26]; Ji et al., 2017[27]; Ju et al., 2018[28]; Kee et al., 2016[29]; Lan et al., 2017[30]; Lazo-Gomez and Tapia, 2017[31]; Li et al., 2018[32]; Liu and Zhou, 2017[34]; Liu et al., 2017[35]; Lu et al., 2018[36]; Maciel et al., 2016[37]; Maksymchuk et al., 2017[38]; Mitani et al., 2017[40]; Mkhize et al., 2017[41]; Naseer et al., 2017[42]; Pandya et al., 2017[45]; Patrizio et al., 2018[46]; Qin et al., 2017[47]; Ren et al., 2018[48]; Sameni et al., 2018[49]; Singh et al., 2018[51]; Sohn et al., 2018[52]; Tinay et al., 2017[53]; Veith et al., 2017[54]; Wu et al., 2018[55]; Xingyu et al., 2016[56]; Yang et al., 2017[57]; Yang et al., 2018[58]; Yarahmadi et al., 2017[60]; Yarahmadi et al., 2018[59]; Yazıcı et al., 2018[61]; Yuan et al., 2016[63]; Yuan et al., 2018[62]; Zhang et al., 2018[64]; Zhao et al., 2017[65]; Zhu et al., 2018[66]).

EXCLI Journal 2018;17:856-863 – ISSN 1611-2156
Received: July 15, 2018, accepted: August 18, 2018, published: August 27, 2018

| Key findings | Reference |
|---|---|
| Quercetin acts as potential agent to reduce oxidative stress likely caused by hyperglycemia and diabetes by modulating some signaling pathways known to be associated with cancer. | Yarahmadi et al., 2018 |
| Owing to its antioxidant property, Quercetin can effectively improve hepatic oxidative damage and hepatotoxicity induced by small-sized gold nanoparticles. | Abdelhalim et al., 2018 |
| Quercetin has therapeutic potential in treatment for renal injury. It can reduce renal injury by modulating macrophage polarization. | Lu et al., 2018 |
| Quercetin has been shown to significantly reduce the acute phase toxic effects of *Echis pyramidum* venom on the liver and kidneys of rats by preventing oxidative stress in these organs. It has anti-inflammatory, anti-edema, anti-hemorrhagic, and PLA2-inhibitory properties, owing to which it might act as a multi-action antidote against snake venom-induced toxicity. | Al-Asmari et al., 2018 |
| Quercetin showed restorative effects on cellular senescence by down-regulating the activities associated with senescence and up-regulating the expression of anti-oxidant enzyme genes in the aged human dermal fibroblasts. | Sohn et al., 2018 |
| Quercetin nanoparticles are better than quercetin for inhibiting cell growth, as they obstruct cell cycle and assist in apoptosis in MCF-7 (Michigan Cancer Foundation-7) cells; therefore, quercetin nanoparticles could be used for the treatment or prevention of breast cancer. | Aghapour et al., 2018 |
| Quercetin-mediated relaxation of the basilar artery *in vitro* is partly dependent on the endothelium, which is mostly related to the pathways of NO and COX. It also influences relaxation through the occlusion of Ca channels. | Yuan et al., 2018 |
| Through a novel mechanism, quercetin was shown to control the secretory function of intestinal goblet cells and mucin levels in the enterocytes, thereby exerting protective effects on the intestinal mucosal barrier. | Damiano et al., 2018 |
| Quercetin might protect the mouse embryo against actinomycin D by increasing the number of viable cells and decreasing the number of apoptotic cells, thereby contributing to the expansion of the blastocysts, reduction in the thickness of zona pellucida, and increase in the hatching rate of embryos. | Sameni et al., 2018 |
| Quercetin obstructs the accumulation of azidothymidine-induced neuroinflammation in the central nervous system via Wnt5s-mediated inhibition. | Yang et al., 2018 |
| Chronic supplementation of quercetin exhibits antioxidant potential prior to and after strenuous sporadic exercise, thus making the erythrocytes competent to better cope with an oxidative insult. | Duranti et al., 2018 |
| The acute ingestion of quercetin enhances neuromuscular performance during and after resistance training sessions. | Patrizio et al., 2018 |
| Quercetin, quercetin-3'-sulfate, and quercetin-3-glucuronide exhibit strong antitumor effects by inducing a reactive oxygen species-dependent apoptotic pathway in MCF-7 cells. | Wu et al., 2018 |
| Quercetin application and low-level laser therapy together was shown to improve wound healing in both non-diabetic and diabetic rats, when compared with their individual effects. | Ahmed et al., 2018 |
| Quercetin, in combination with low-levels of doxorubicin, showed anti-tumor activity and acted as a novel agent for breast cancer therapy, and also attenuated doxorubicin's toxic side effects. | Li et al., 2018 |
| Quercetin, along with green tea, actuates signaling pathways related to apoptosis, cell cycle, and autophagy; these signaling pathways act in concert to generate antigrowth effects in HL-60 xenograft in mice, indicating that these compounds have the ability to act as allies in cancer treatment. | Calgarotto et al., 2018 |
| Both Quercetin and ellagic acid are used to reduce hepatotoxicity through their antioxidant, metal-chelating, and anti-inflammatory effects. | Afifi et al., 2018 |
| Quercetin inhibits xanthine oxidase, and therefore, has been used as a potential nutritional supplement for protecting against gout and peroxidative damage. | Zhang et al., 2018 |

EXCLI Journal 2018;17:856-863 – ISSN 1611-2156
Received: July 15, 2018, accepted: August 18, 2018, published: August 27, 2018

| Key findings | Reference |
| --- | --- |
| The combined application of quercetin and insulin not only overcomes poor oral bioavailability, but also averts the generation of reactive oxygen species responsible for diabetes-mediated complications. | Singh et al., 2018 |
| Quercetin induces the up-regulation of scavenger receptor class B type I and successive lipid uptake in the hepatocytes, suggesting its beneficial effects on cholesterol homeostasis and atherogenesis. | Ren et al., 2018 |
| A diet having either 0.1 or 1 % quercetin enhances the antitumor effect of tri-chostatin A (TSA), as well as prevents TSA-induced muscle wasting. | Chan et al., 2018 |
| From both *in vivo* and *in vitro* trails, it has been shown that Quercetin impedes the development of renal cyst *in vitro* and *in vivo*, affirming a novel candidate strategy for autosomal dominant polycystic kidney disease treatment. | Zhu et al., 2018 |
| Quercetin has been used to promote antioxidant defense mechanisms and prevents oxidative stress, intestinal damage, and necrotizing enterocolitis development. | Yazıcı et al., 2018 |
| Quercetin could reinstate the actual intestinal host-microbe relationship to improve colitis by restoring the pro-inflammatory, anti-inflammatory, and bactericidal functions of enteric macrophages. | Ju et al., 2018 |
| Quercetin modulates γ-enolase expression in response to cerebral ischemia, indicating its neuroprotective effect. | Jeon et al., 2017 |
| Quercetin impedes adipogenesis in muscle satellite cells *in vitro* by controlling the transcription of adipogenic markers. | Funakoshi et al., 2017 |
| Inhalation treatment with quercetin helps in alleviating radiation-induced pneumonitis by decreasing inflammatory cell number and attenuating inflammatory response and pathological changes. | Qin et al., 2017 |
| Quercetin shows highly significant reduction in ulcer size when compared with the effects of benzydamine hydrochloride. Quercetin application is a safe, well-tolerated, and effective therapy that promotes complete ulcer healing within a short period. | Pandya et al., 2017 |
| Quercetin induces apoptosis in Y79 cells by stimulating the pathways of c-Jun N-terminal kinase and p38 mitogen-activated protein kinase, providing a novel treatment approach for human retinoblastoma. | Liu and Zhou, 2017 |
| Quercetin restores the body's sensitivity to corticosteroids, and therefore, might be used as a potential therapeutic agent in combination with corticosteroids in a novel treatment strategy for chronic obstructive pulmonary disease. | Mitani et al., 2017 |
| Quercetin exerts its beneficial effects through a sirtuin 1-mediated mechanism. Sirtuin 1 plays an important role in excitotoxic neurodegeneration, and therefore, its pharmacological modulation might provide opportunities for therapy in motor neuron disorders. | Lazo-Gomez and Tapia, 2017 |
| A combination of quercetin and resveratrol showed beneficial effects in alleviating obesity due to high-fat diet (HFD) and decreasing HFD-induced gut microbiota dysbiosis. | Zhao et al., 2017 |
| CYP2E1 has a vital role in stress-induced pathological processes in diabetic liver; however, quercetin inhibits the secretion of enzymes during the development of diabetes, thereby preventing oxidative damage in the liver. | Maksymchuk et al., 2017 |
| Quercetin treatment could prevent the formation of abnormal human bone in lipopolysaccharide-induced bone diseases. | Guo et al., 2017 |
| Quercetin reduces the symptoms of idiopathic pulmonary fibrosis by restoring the disturbed redox balance and reducing inflammation. | Veith et al., 2017 |
| Quercetin might have a therapeutic potential in treating human osteosarcoma because of its inhibitory effects on cell migration and invasion. | Lan et al., 2017 |

| Key findings | Reference |
|---|---|
| Quercetin decreases the rate of hyperalgesia caused by tamoxifen-induced adenomyosis in mice. The mechanism of the above-mentioned response is the reduction of central sensitization, which could provide a promising therapy for adenomyosis. | Nie and Liu, 2017 |
| Through its anti-inflammatory effects, Quercetin reduces neuropathic pain by inhibiting the Toll-like receptor signaling pathway. | Ji et al., 2017 |
| Quercetin protects mice from both d-galactose-induced cognitive functional impairment and neuronal cell apoptosis through initiation of the Nrf2-ARE signaling pathway. | Dong et al., 2017 |
| Quercetin exerts a protective effect on vascular calcification in adenine-induced chronic renal failure rats, possibly through the intonation of oxidative stress and induction of the nitric oxide synthase (iNOS)/p38 mitogen-activated protein kinase (p38MAPK) pathway. | Chang et al., 2017 |
| Quercetin, when used in association with different levels of pesticides (5 mg/kg) or their mixture (10 mg/kg), prevents mitochondrial swelling in varied brain tissues. | Beghoul et al., 2017 |
| Quercetin and melatonin are beneficial against necrotic and apoptotic cell death during steatosis. Thus, both agents might be beneficial in the improvement of hepatic steatosis in association with conventional therapy in humans | Esrefoglu et al., 2017 |
| Quercetin inhibits microglia-mediated inflammatory response through the induction of heme oxygenase-1, thereby protecting against obesity-induced hypothalamic inflammation. | Yang et al., 2017 |
| Nicotine-induced imbalance between the production of free radicals and the functioning of the antioxidant defense systems could be prevented using quercetin, thereby suggesting that the administration of this potent antioxidant might be a good option in clinical applications where cellular damage is a consequence of reactive oxygen species. | Yarahmadi et al., 2017 |
| Quercetin acts against ischemia reperfusion injury by inducing heme oxygenase-1 (HO-1) and by exhibiting an unexpected hepatoprotective effect. Tin protoporphyrin (SnPP) also showed similar effects. Because of its superior ability to induce HO-1, quercetin has a more prominent protective effect than SnPP. | Atef et al., 2017 |
| Quercetin shows the ability to reduce prenatal stress-induced pro-inflammatory marker (interleukin 1 beta) levels, thereby impairing febrile seizures. Therefore, quercetin could be used as a therapeutic agent for treating febrile seizures in prenatally stressed individuals. | Mkhize et al., 2017 |
| Quercetin treatment improves follicular development, reduces granulosa cell apoptosis, and upholds oocyte competence in heat-stressed rabbits. | Naseer et al., 2017 |
| Quercetin has a potential clinical application for treating glioblastoma because of its inhibitory effects on glioblastoma cell proliferation and invasion. | Liu et al., 2017 |
| Quercetin enhances functional recovery by up-regulating neuronal intrinsic growth capacity and postponing distal atrophy. In general, quercetin activates multiple effects to uphold behavioral revival following sciatic nerve-crush injury in mice. | Chen et al., 2017 |
| Quercetin application was demonstrated to be effective for pulmonary arterial hypertension probably because it inhibits endothelial trans differentiation possibly via modulating the expression of Akt and Erk1/2. | Huang et al., 2017 |
| Quercetin treatment secures the contractility of bladder tissue against acute ischemia/reperfusion (I/R) injury by reducing the oxidative stress and apoptosis induced by I/R. | Tinay et al., 2017 |
| Quercetin inhibits the survival and metastatic ability of CT26 (colon 26) cells, suggesting that it suppresses colorectal metastasis in a mouse model. These results designate quercetin as a potential therapeutic agent for the treatment of metastatic colorectal cancer. | Kee et al., 2016 |

| Key findings | Reference |
|---|---|
| By inhibiting oxidative stress, quercetin suppressed cadmium-induced autophagy. This finding provides a theoretical basis for the treatment of cadmium injury. | Yuan et al., 2016 |
| Injection of quercetin in A549 tumor-bearing mice effectively reduced cancer cell growth *in vivo*. Histone 3 phosphorylation decreased in tumor tissues after quercetin treatment. Thus, quercetin can suppress lung cancer cell growth *in vitro* and *in vivo* as an aurora B inhibitor. | Xingyu et al., 2016 |
| Quercetin prevents the impairment of enzymes regulating purinergic and cholinergic extracellular signaling and improves memory and anxiety-like behavior, as seen in an animal model of streptozotocin-induced diabetes. | Maciel et al., 2016 |
| Quercetin was shown to protect liver function against acrylamide-induced DNA damage in rats. | Ansar et al., 2016 |
| Quercetin triggers the endoplasmic reticulum stress (ERS) signaling pathway in hepatic stellate cells, leading to apoptosis. The research group identified an ERS-mediated mechanism of action for quercetin as a promising antifibrotic agent. | He et al., 2016 |

## Conflict of interest

The authors declare no conflict of interest.

## Acknowledgements

This work was supported by a grant from the Next-Generation BioGreen 21 Program (SSAC, Project #. PJ013328)" Rural Development Administration, Republic of Korea.

## Conflict of interest

The authors declare no conflict of interest.

## Figures and Tables

**Table 1:**
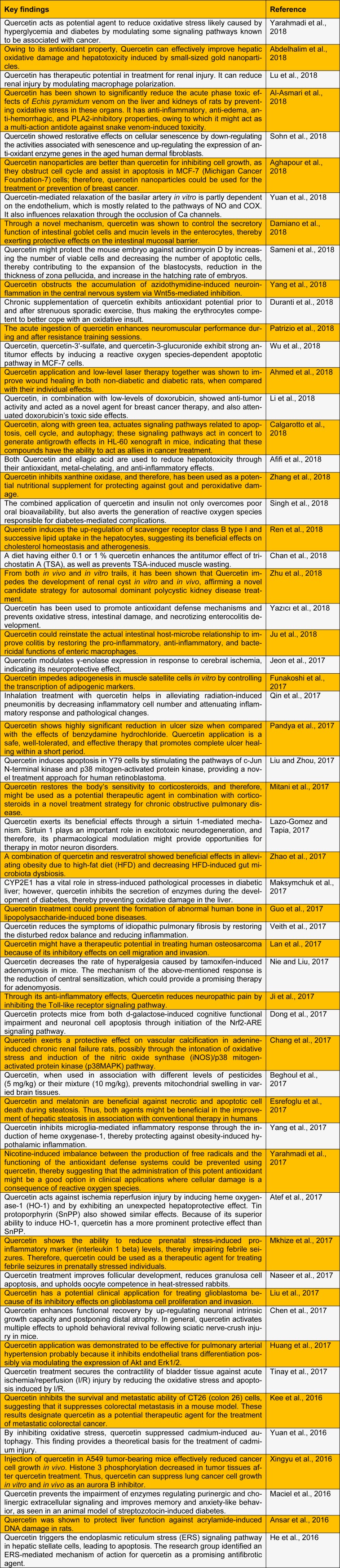
Recent studies of the biological and pharmacological activities of quercetin
